# Mitochondria-Targeted Antioxidants: Future Perspectives in Kidney Ischemia Reperfusion Injury

**DOI:** 10.1155/2016/2950503

**Published:** 2016-05-24

**Authors:** Aleksandra Kezic, Ivan Spasojevic, Visnja Lezaic, Milica Bajcetic

**Affiliations:** ^1^School of Medicine, University of Belgrade, Dr. Subotica 8, 11000 Belgrade, Serbia; ^2^Clinic for Nephrology, Clinical Center of Serbia, Pasterova 2, 11000 Belgrade, Serbia; ^3^Department of Life Sciences, Institute for Multidisciplinary Research, University of Belgrade, Kneza Vieslava 1, 11000 Belgrade, Serbia; ^4^Department of Pharmacology, Clinical Pharmacology and Toxicology, School of Medicine, University of Belgrade, P.O. Box 38, 11000 Belgrade, Serbia; ^5^Clinical Pharmacology Unit, University Children's Hospital, 11000 Belgrade, Serbia

## Abstract

Kidney ischemia/reperfusion injury emerges in various clinical settings as a great problem complicating the course and outcome. Ischemia/reperfusion injury is still an unsolved puzzle with a great diversity of investigational approaches, putting the focus on oxidative stress and mitochondria. Mitochondria are both sources and targets of ROS. They participate in initiation and progression of kidney ischemia/reperfusion injury linking oxidative stress, inflammation, and cell death. The dependence of kidney proximal tubule cells on oxidative mitochondrial metabolism makes them particularly prone to harmful effects of mitochondrial damage. The administration of antioxidants has been used as a way to prevent and treat kidney ischemia/reperfusion injury for a long time. Recently a new method based on mitochondria-targeted antioxidants has become the focus of interest. Here we review the current status of results achieved in numerous studies investigating these novel compounds in ischemia/reperfusion injury which specifically target mitochondria such as MitoQ, Szeto-Schiller (SS) peptides (Bendavia), SkQ1 and SkQR1, and superoxide dismutase mimics. Based on the favorable results obtained in the studies that have examined myocardial ischemia/reperfusion injury, ongoing clinical trials investigate the efficacy of some novel therapeutics in preventing myocardial infarct. This also implies future strategies in preventing kidney ischemia/reperfusion injury.

## 1. Introduction

Ischemia/reperfusion injury (IRI) is a major cause of acute kidney injury (AKI) formerly known as acute renal failure [1]. The incidence of AKI in hospitalized patients has been reported to be between 2% and 7% and even greater than 10% in intensive care unit (ICU) patients contributing to increased mortality rate [2]. Kidney IRI is of great importance occurring in various clinical settings including shock, vascular and cardiac surgery, sepsis, and kidney transplantation. During kidney transplantation, IRI causes delayed graft function (DGF) that has been associated with more frequent episodes of acute rejection and progression to chronic allograft nephropathy [2–4]. Complex interplay of pathophysiological processes linking inflammation, abnormal repair, and fibrosis makes AKI an important risk factor for progression of chronic kidney disease [5–7]. Basically, reperfusion phenomena consist of events which “paradoxically” continue to damage tissue in spite of established circulation and oxygen supply to the tissue that previously was under ischemia. Pathogenesis of IRI is rather complex and involves hypoxic injury, production of reactive oxygen species (ROS), inflammation, apoptosis, and necrosis [8]. Reactive oxygen species (ROS) include oxygen radicals such as superoxide radical anion (O_2_^∙−^) and hydroxyl radical (HO^∙^) and certain nonradicals that either are oxidizing agents or are easily converted into radicals, such as hydrogen peroxide (H_2_O_2_) and hypochlorous acid (HOCl). ROS generation represents a cascade of reactions starting with the production of O_2_^∙−^ that can be further converted to H_2_O_2_ via superoxide dismutases (SOD), manganese (MnSOD) in mitochondria and copper-zinc (CuZnSOD) in the cytosol. The main sinks for H_2_O_2_ are catalase (CAT) and glutathione peroxidase (GPx). The latter uses glutathione (GSH) which is oxidized to GSSG and recycled by glutathione reductase. There are other enzymes that can remove H_2_O_2_, such as peroxiredoxin/thioredoxin/thioredoxin reductase (Prx/Trx/TrxR) system. However, CAT activity is about three orders of magnitude higher compared to Prx/Trx/TrxR system [9], which is essential under physiological settings for keeping low levels of mitochondrial H_2_O_2_ emission and for normal redox signaling via regulation of thiol redox switches on different proteins [10].

H_2_O_2_ can also react with transition metals, such as iron or copper, to produce HO^∙^, the most reactive species in living systems [11]. The main reactive nitrogen species (RNS) are nitric oxide (NO^∙^) and peroxynitrite (ONOO^−^). ONOO^−^ is formed via reaction between NO and O_2_^∙−^ and can be further protonated and decomposed to nitrogen dioxide radical (NO_2_^∙^) and HO^∙^ [12, 13]. These radicals are “caged” (i.e., generated close to each other), so they can recombine quickly, and much of ONOO^−^ undergoes isomerisation to nitrate. Some amount of ONOO^−^ *in vivo* reacts with CO_2_ to form nitrosoperoxycarbonate (ONOOCO_2_^−^). About 35% of ONOOCO_2_^−^ is decomposed to NO_2_^∙^ and carbonate radical (CO_3_^∙−^) [14]. The latter is highly oxidizing species targeting NADPH and proteins [15, 16].

Mitochondria are the major site of ROS production, due to inevitable leakage of electrons from electron transport chain (ETC) onto oxygen [17]. Other major intracellular sites of ROS generation are enzymes, such as NADPH oxidase (NOX) and xanthine oxidase (XO).

Initially, in ischemic phase kidney tubular epithelial and endothelial cells are main producers of ROS and are later accompanied by activated leucocytes, that is, oxidative burst related to inflammation. These events reveal the role of ROS in exerting detrimental effects on cellular structure, linking oxidative stress, inflammation, and cell death. ROS through interactions with small metabolites as well as proteins, lipids, and nucleic acids might irreversibly destroy or alter the function of these target molecules and belonging organelles and cells. ROS can also serve as homeostatic signaling molecules which primarily depends on magnitude and duration of provoking stimuli for ROS production.

In recent years it has become clear that mitochondria have critical role in initiation and progression of renal IRI. They are early responders to the anoxia and then reoxygenation initiating responses that lead to changed metabolic and bioenergetic status, autophagy, inflammation, and induction of cell death pathways. Several approaches, mainly in experimental studies with a few human trials, have been used in investigating the options for preventing and treatment of IRI with special emphasis on modulation of inflammatory response, inhibition of apoptosis, and amelioration of oxidative stress, but currently there is no effective pharmacological treatment to address the main mechanisms of ischemic AKI [18–20]. Recently, novel therapeutic approach called ischemic preconditioning has become translated from animal models to humans [20, 21]. It is based on the observations that episodes of nonlethal ischemia can precondition the kidney to be protected in subsequent prolonged ischemia.

Nevertheless, oxidative stress is crucial for the cascade of processes participating in the pathogenesis of IRI. Since mitochondria as both sources and targets of ROS are initiators of complex mechanisms in IRI, it seems reasonable from therapeutic perspective to develop pharmacological method aiming to decrease mitochondrial oxidative damage.

In this review, we will summarize the mechanisms of mitochondrial ROS production and some options for potential treatment strategies.

## 2. The Role of Mitochondria in the Pathogenesis of IRI

**Figure 1:**
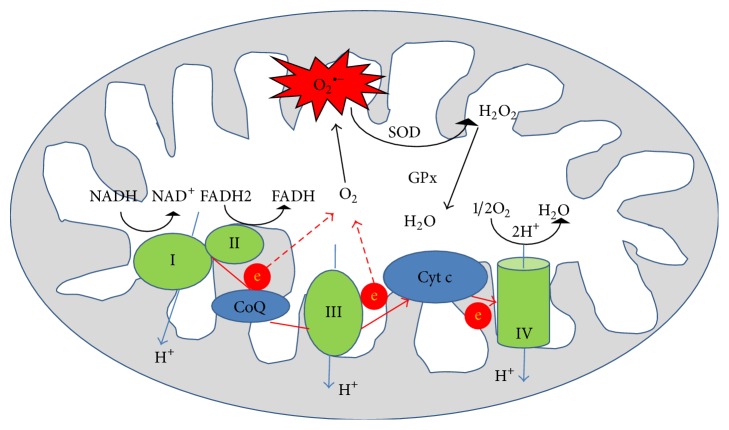
Formation of various reactive oxygen species from electron transport chain in mitochondria. O_2_^∙−^: superoxide anion; SOD: superoxide dismutase; GPx: glutathione peroxidase; CoQ: coenzyme Q; Cyt c: cytochrome c.

By impairing electron transport and energy metabolism and by altering cellular redox potential via ROS production, mitochondria trigger events leading to apoptosis, a hallmark of IRI. Electron transport along ETC to O_2_ is tightly coupled to oxidative phosphorylation for ATP synthesis. In normal, for instance, nonischemic cells, the main source of ROS is ETC [22, 23]. Most oxygen consumed is reduced to water through 4 steps of single electron reduction by cytochrome c oxidase. Electrons generated from reduced nicotinamide adenine dinucleotide (NADH) are accepted by NADH dehydrogenase (Complex I) and those from succinate are accepted by succinate dehydrogenase (Complex II). Electrons are then passed to cytochrome bc1 (Complex III) through coenzyme Q (CoQ) and to cytochrome c oxidase (Complex IV) using cytochrome c as a carrier. The last step is transfer of electron from cytochrome c oxidase to O_2_ to form water (Figure 1). Electron flow mediated by the respiratory chain enzyme complex drives proton (H^+^) translocation from the matrix side at the level of complexes I, III, and IV to the intermembrane space side, thereby establishing an electrochemical potential gradient or proton motive force across the inner membrane [23]. Since the inner mitochondrial membrane is almost impermeable, this electrochemical gradient is used to reintroduce protons back through the proton channel of complex V (ATP synthase). ATP synthesis from ADP and inorganic phosphate is then catalyzed by F_0_F_1_ATPase. Every decrease in the rate of mitochondrial phosphorylation increases electron leakage from the ETC and consequently increases the production of O_2_^∙−^. Mitochondria have defense mechanism to neutralize ROS. Superoxide radical anion is converted to H_2_O_2_ by MnSOD, and H_2_O_2_ is further degraded to H_2_O. In mitochondria, about 70–80% of H_2_O_2_ removal has been attributed to GPx [9]. ROS may induce mild mitochondrial oxidative stress or diffuse to the cytosol playing an important role in cellular homeostasis, mitosis, and differentiation and serving as signaling molecules in different physiological responses [24, 25]. This constant production of small amounts of ROS is necessary to maintain the appropriate “redox state” of cell which is crucial for the activation of several genes and the function of numerous enzymes. On the other hand, H_2_O_2_ that escapes mitochondria is removed by CAT, GPx, and other H_2_O_2_ removing systems, but an excess can activate potentially detrimental cascades, for example, via NF-*κ*B.

What happens to the mitochondria in ischemia? The effect depends on the duration of ischemia. Ischemia causes alterations to the mitochondrial ETC complexes. If ischemic episodes are of short duration the electronegativity of the ETC complexes and leakage of electrons is increased with consequently increased ROS formation. ROS can trigger signaling events that lead to the synthesis of proteins, including MnSOD and thereby providing beneficial role that is the part of ischemic preconditioning phenomena [26]. A prolonged period of ischemia results in decreased activity of the complexes I and IV of ETC and subsequent electron leak of that reduce O_2_ to form superoxide radicals when O_2_ is reintroduced following reperfusion [24, 27]. Also, impaired ETC results in decreased ATP production following reperfusion. During prolonged ischemia more of detrimental radicals are produced. Superoxide mainly attacks Fe-S centers in ETC proteins to provoke the reduction of Fe^3+^ and liberation of Fe^2+^, leaving aconitase and other transporters of electrons dysfunctional [28]. These Fe^2+^ ions are important in Fenton chemical reaction whereby H_2_O_2_ can be converted to the highly reactive HO^∙^ which is more detrimental for cell structure proteins and membrane lipids. It is important to note that HO^∙^ has a drastically shorter diffusion radius in physiological milieu compared to H_2_O_2_, and it is too reactive to pass membranes. In short, HO^∙^ is more dangerous for targets that are nearby the site of production. H_2_O_2_ generated in mitochondria can affect other organelles, nucleus, and surrounding cells [29]. The prolonged ischemia decreases the activity of antioxidant enzymes, such as MnSOD, and causes GSH depletion [30, 31].

The involvement/interference of ROS in signaling cascades might have both detrimental and beneficial effects. Cellular hypoxia appears to be the key signal for activation of HIF-1*α*, nuclear factor-*κ*B (NF-*κ*B), activator protein 1 (AP-1), and mitogen activated protein kinases (MAPK). In addition, ROS have been directly implicated in programmed cell death [2, 32, 33]. Under hypoxia, transcriptional cell activity is directed to synthesis of proinflammatory and cytoprotective molecules [34]. So far the available data have implied a proinflammatory action of NF-*κ*B as one of the key players in pathogenesis of IRI [35–38]. Besides chemokines and cytokines, NF-*κ*B is implicated in the production of both ROS and HIF-1; that is, there is a positive feedback loop serving as an amplification mechanism [35, 39].

Massive production of ROS during reperfusion is secondary to electron leak mostly at complexes I and III [40]. The overproduction of O_2_^∙−^ may lead to formation of ONOO^−^ which is a highly reactive molecule and leads to nitration of proteins, including complexes I and III, and further tissue injury [40, 41]. Renal content of 3-nitrotyrosine (the footprint of peroxynitrite) increases during ischemia. Pertinent to this, ^∙^NO that is formed via activity of inducible NO synthase (iNOS) is also increased [42–44]. iNOS is induced in kidney IRI [45–48]. Studies using inhibition of expression and activity of iNOS or even absence of iNOS showed amelioration of kidney IRI suggesting that NO generated by iNOS had detrimental role and contributed to kidney IRI [45, 47, 49]. This, by oxidant-induced disruption of protein complexes I and III, potentiates electron leak and further O_2_^∙−^ generation. The whole process is driven by ROS-induced ROS release and may become a vicious cycle that induces mitochondrial permeability transition pore (mPTP) opening [50]. Because of mitochondrial GSH depletion during ischemia, conversion of H_2_O_2_ to water is insufficient in reperfusion favoring formation of HO^∙^ via Fenton reaction [24, 31]. As a consequence, membrane permeability is affected. “Redox state” is altered by the oxidation of pyridines and thiols with consequent modification of NADH/NAD^+^ and GSH/GSSG ratio [44]. Excessive ROS formation, recovery of pH, and calcium overload facilitate the opening of mPTP with consequent loss of cytochrome c and pyridine nucleotides favoring further ROS generation and triggering cell death [50–52]. The opening of mPTP results in redistribution of NADH and calcium to the cytosol and an influx of water to mitochondria causing mitochondrial matrix swelling and outer mitochondrial membrane rupture with release of proapoptotic factors leading to cell death. Released calcium to cytosol activates proteases, nucleases, and phospholipases, which trigger apoptosis [52].

Additionally, mitochondria has other components besides ETC that contribute to ROS production including NOX4, monoamine oxidase, and growth factor adaptor protein, Shc (p66^Shc^), but this contribution is rather low compared with the generation of ROS from ETC [22].

## 3. Mode of the Action of Mitochondria-Targeted Drugs

According to previously mentioned data, it seems reasonable to develop pharmacological method that decreases mitochondrial oxidative damage in order to decrease kidney IRI. The relatively unsatisfactory efficacy of conventional antioxidants may be the consequence of their low penetrance to the mitochondria interior, which not only is the main site of ROS production but also suffers from oxidative stress as other cellular compartments. The inner mitochondrial membrane is highly impermeable and rich in cardiolipin and maintains a strong negative internal potential of −180 mV that is required for the function of electron transport chain.

To overcome these limitations, mitochondria-targeted antioxidants have been developed to provide their delivery to the mitochondrion interior. Mitochondria-targeted antioxidants are usually chimeric molecules of a cation triphenylphosphonium (TPP) conjugated with an antioxidant moiety such as coenzyme Q_10_ or plastoquinone [53, 54]. The proton motive force in the inner mitochondrial membrane maintaining the large mitochondrial membrane potential and the positive charge of lipophilic cation drive a transport of these cationic antioxidants into mitochondria. The result of this mitochondrial uptake is a chimeric drug concentration 10,000 times higher in the mitochondrial matrix than in the cytosol [55]. Apart from the TPP, rhodamine 123 is another suitable lipophilic cation to be conjugated to mitochondria-selective molecules [56]. However, lipophilic cations have a disadvantage. Since the charge accumulation into the matrix leads to mitochondrial membrane depolarization, at concentrations greater than 10 *μ*M, toxicity has been observed [56].

Recently, use of short peptide sequences with specific physicochemical properties for delivery of compounds to inner mitochondria has emerged [57]. The Szeto-Schiller SS peptides have exhibited marked antioxidant properties by scavenging ROS and inhibiting linoleic acid oxidation [58, 59]. They feature a common structural motif of alternating aromatic (Phe, Tyr, and Dmt (2′,6′-dimethyltyrosine)) and basic (Arg, Lys) residues. SS peptides freely penetrate membranes in a potential-independent manner due to their aromatic-cationic amino acid sequence. Tyr or Dmt residues are likely responsible for the ROS scavenging abilities of these peptides [60]. Another optional oligopeptide is conjugated to manganese metalloporphyrin and belongs to novel class of mitochondria-targeted SOD mimics named Mn-porphyrin-oligopeptide conjugates [61].

*N*-acetyl-L-cysteine (NAC) has been known for the efficiency in protecting cells against oxidants [62]. In order to deliver the tripeptide glutathione (L-*γ*-glutamyl-L-cysteinylglycine or GSH) and its analog NAC into mitochondria, choline esters (MitoGSH and MitoNAC) have been utilized [63]. Experiments with MitoNAC were performed using cultured cells, However,* in vivo* data are missing.

Among the other mitochondria-targeted compounds with different mode of penetrance and action that are worth mentioning is diazoxide, the opener of mitochondrial K_ATP_ channels. The precise mechanisms how active mitochondrial K_ATP_ channels lead to decreased ROS production when oxygen is delivered during reperfusion are still unclear, although they are in some ways similar to events elicited by ischemic preconditioning [64]. Opened K channels result in a lowered mitochondrial membrane potential and lowered redox state of NAD system leading to decreased ROS production by respiratory chain Complex I [65].

## 4. Mitochondria-Targeted Antioxidants in Renal IRI

**Figure 2:**
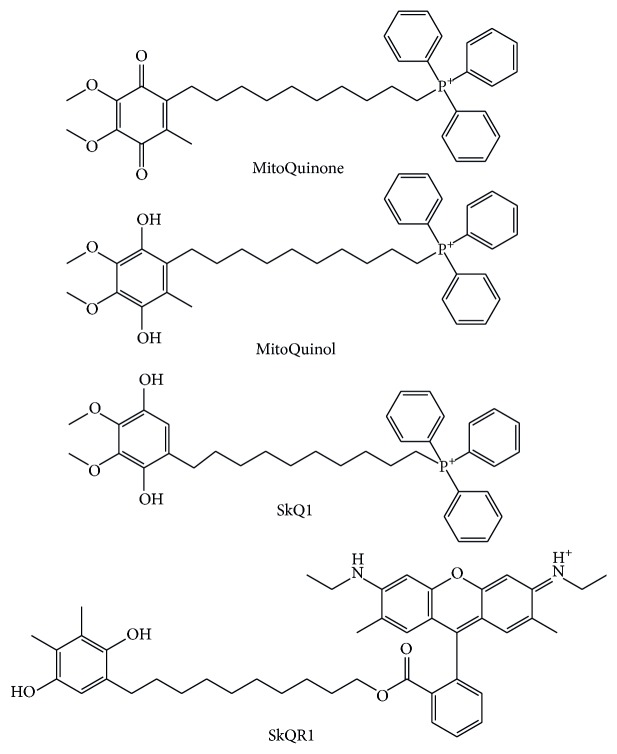
Mitochondria-targeted antioxidants with ubiquinone or plastoquinone antioxidant moiety, that are used in kidney ischemia/reperfusion injury.

Mitochondria-targeted antioxidants have already been used in other experimental pathology models and one of them, MitoQ, has been used in two Phase II trials in humans regarding treatment of Parkinson disease and chronic hepatitis C, showing long term safety and tolerance [66, 67]. Because of a positively charged lipophilic cation, MitoQ is accumulated in the negatively charged interior of mitochondria. The antioxidant component of MitoQ is the ubiquinone that is also found in coenzyme Q_10_ (Figure 2) [53]. By the action of the enzyme Complex II in the mitochondrial respiratory chain, ubiquinone part of MitoQ is rapidly activated to the active ubiquinol antioxidant [68]. After detoxifying ROS, the ubiquinol part of MitoQ is converted to ubiquinone, which is again subjected to Complex II to be recycled back to active antioxidant ubiquinol [68]. This process makes MitoQ an effective mitochondria-targeted antioxidant.

The reason for the use of MitoQ in kidney IRI came from the studies using MitoQ to decrease heart and hepatic IRI and to prevent kidney damage during cold storage [69–71]. Using this model, it was demonstrated that administration of MitoQ prior to the onset of ischemia reduced oxidative damage and severity of renal IRI, thereby providing functional protection to the kidney [72]. The group of Skulachev et al. synthesized plastoquinonyl-decyl-triphenylphosphonium. In this compound named SkQ1, ubiquinone was replaced by plastoquinone (Figure 2) [54, 73]. The effect of SkQ1 on kidney IRI using a culture of kidney epithelial cells has been studied. Preincubation with SkQ1 increased survival of these cells and diminished mitochondrial fission induced by the anoxia/reoxygenation procedure [74, 75]. In experimental model of a single-kidney ischemia (90 min) followed by reoxygenation, injection of SkQ1 to rats a day before kidney ischemia significantly improved survival compared to the experimental animals subjected to kidney IRI without any previous treatment [74].

In another experimental study, the authors used a mitochondria-targeted compound containing a charged rhodamine molecule conjugated with plastoquinone named SkQR1 (Figure 2) [76]. An intraperitoneal injection of SkQR1 to rats exposed to kidney IRI normalized the ROS level and lipid peroxidized products in kidney mitochondria, significantly decreased BUN and blood creatinine and lowered mortality compared to animals that were subjected to kidney IRI without any given drug [76]. Additionally, SkQR1 was found to provide the kidney with elements of ischemic tolerance signaling mechanisms. Administration of SkQR1 induced erythropoietin (EPO) and the phosphorylated form of GSK-3*β* in rat kidney [76]. EPO is known to afford some protection against ischemic damage [77, 78]. In a pilot clinical trial investigating the efficacy of EPO to prevent AKI after coronary artery bypass grafting (CABG), it was shown that EPO administration resulted in an incidence of AKI of 8% compared with an incidence of 29% in the placebo group [79]. Phosphorylated form of GSK-3*β* correlates with activity of prosurvival genes. Via inhibition of GSK-3*β*, protective signaling pathways act on the end effector mPTP; that is, they prevent the induction of the mitochondrial permeability transition, restore mitochondrial membrane potential, and decrease ROS production [80–82]. The authors concluded that treatment with SkQR1 provided linked synergy between antioxidative effects and ischemic tolerance signaling mechanisms due to the targeted delivery of this compound to mitochondria [76]. Beside the kidney IRI, MitoQ and SkR1 have been shown to have the role in amelioration of AKI induced by cisplatin and gentamycin [83, 84].

A number of reports demonstrate that open mitochondrial K_ATP_ channels, which are redox sensitive, effectively block the generation and release of ROS [85, 86]. Interestingly, agents opening the ATP-sensitive K channel (K_ATP_) including diazoxide were effective in ameliorating cardiac and neural IRI [87, 88], but ineffective and even injurious in kidney IRI [89]. In a report of Sun et al., a large dose of diazoxide given before kidney ischemia prevented ROS accumulation in mitochondria, thereby reducing oxidative stress, consequent tubular cell apoptosis, and increase in serum creatinine [90]. Considering this effect dependence on widespread opening of the mitochondrial K_ATP_ channels, a large dose of diazoxide is needed, but this phenomenon raises the question of the role of mitochondrial K channels or associated respiratory chain components in the ROS production, depending on specific tissue.

mPTP as another potential target for pharmacological intervention in IRI was supported by preclinical and clinical studies [91, 92]. The substances that block opening of mPTP protect mitochondrial structure and respiration during early reperfusion and accelerate recovery of ATP. CypD is a component of mPTP. Animals with CypD gene ablation or treated with cyclosporin A (CsA), a CypD inhibitor, were protected from renal IRI [93–95]. These promising results were not translated into clinical nephrology compared to extensive investigation in cardiology since the nephrotoxic profile of CsA makes this drug unsuitable for clinical application in the treatment of renal IRI [96, 97].

**Figure 3:**
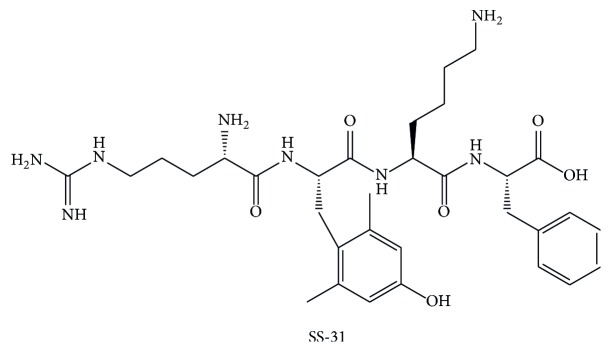
Chemical structure of SS-31.

A growing number of publications speak in favor of compound Szeto-Schiller- (SS-) 31 peptide also known as Bendavia (Figure 3). Besides scavenging of ROS, Bendavia also inhibits mPTP and thereby prevents mitochondrial release of cytochrome c and consequently apoptosis [98]. However, recent study of Brown et al. did not confirm the role of Bendavia in direct inhibition of mPTP but rather emphasized its role in decreased ROS production and indirectly decreased mPTP opening. Bendavia may optimize the mitochondrial phospholipid cardiolipin microdomains, resulting in reduced electron leak from the inner membrane [99].

SS-31 peptide, that is, Bendavia, demonstrated efficacy in several animal models of different pathologic conditions by reducing oxidative stress [100–102]. Regarding IRI, the efficacy of Bendavia was mostly investigated in experimental models of myocardial ischemia. If Bendavia was given to animals in minutes prior to reperfusion or in the first 10 minutes of reperfusion, then the effect of reduced infarction and the extent of coronary no-reflow were achieved [103, 104]. These encouraging experimental results need confirmation in ongoing clinical trials for acute coronary syndromes [105]. Also, in experimental model of kidney, IRI Bendavia reduced oxidative stress, prevented tubular apoptosis and necrosis, and reduced inflammation [106]. Because of the rapid ATP recovery after Bendavia administration, microvascular endothelial cells are protected leading to reduced microvascular congestion that provides better reflow to the medulla [106]. The effect of Bendavia was explored in experimental model of percutaneous transluminal renal angioplasty (PTRA), a condition that may be associated with impairment of renal function. Infusion of Bendavia at the time of percutaneous transluminal renal angioplasty, decreases oxidative stress, apoptosis, and inflammation and improves renal function in animal experimental model of renal artery stenosis [107].

Since oxidative stress is dependent on both ROS production and removal by antioxidant enzymes and taking into consideration that IRI significantly reduces Mn SOD and Cu/Zn SOD mRNA expression, the use of antioxidative enzymes might alleviate kidney IRI. MnSOD knockout (KO) mice that exhibit low expression and activity of MnSOD in distal nephron, after ischemia/reperfusion, show similar levels of injury to the proximal tubule and distal nephron and equally altered renal function when compared with wild-type mice [108]. Additionally, these KO mice exhibit increased proliferating cell nuclear antigen- (PCNA-) positive nuclei in the distal nephrons, autophagy, and mitochondrial biogenesis indicating that chronic oxidative stress stimulates multiple survival signaling pathways to protect kidney against acute oxidative stress following ischemia/reperfusion [108]. The limitation of this study is that proximal tubule, especially its S3 fragment, damaged in IRI the most, in this specific mouse strain was not affected by lack of MnSOD and therefore failed to assess the role of abolished MnSOD activity within proximal tubules in IRI.

**Figure 4:**
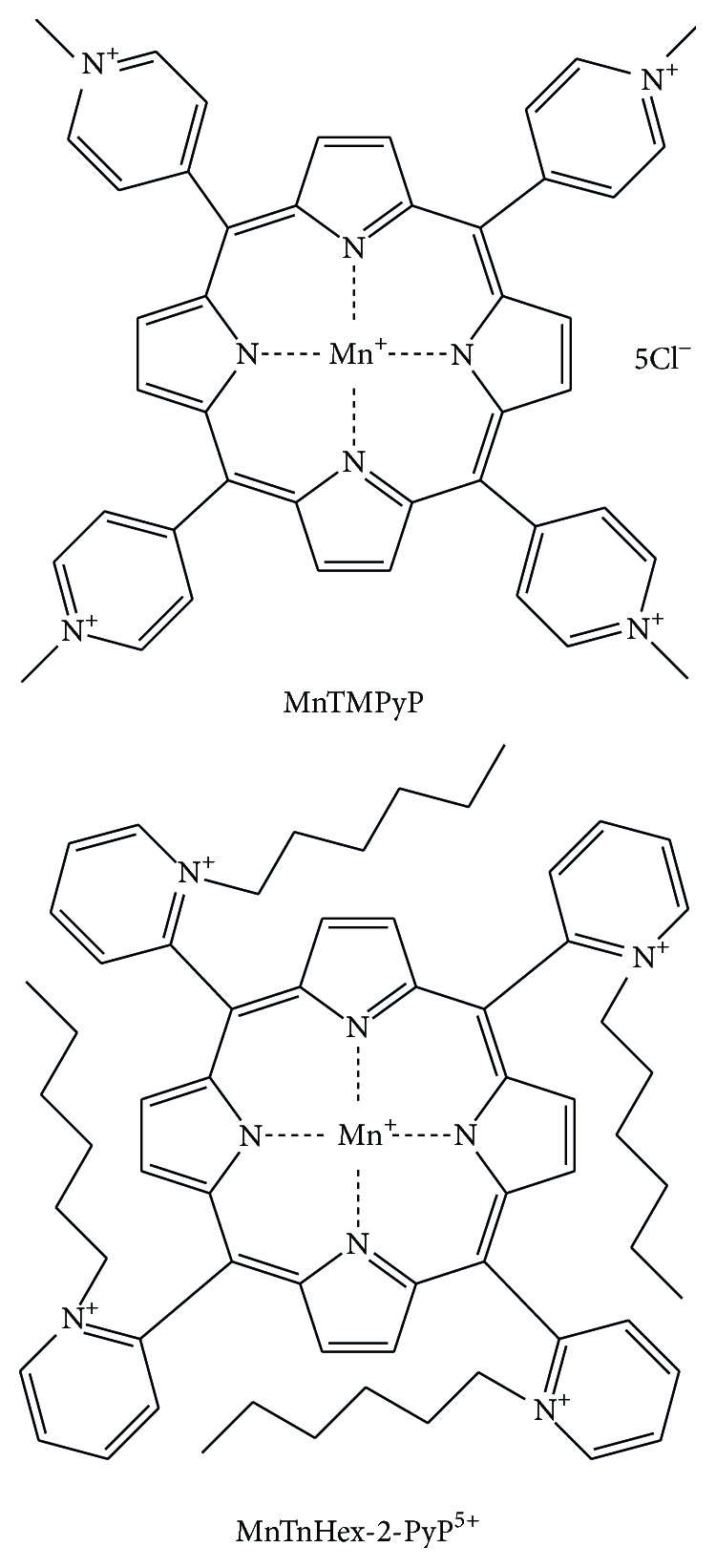
Chemical structures of Mn porphyrins investigated in experimental models of kidney ischemia/reperfusion injury.

There are several reports showing protective role of SOD mimics in experimental models of kidney IRI [109–113]. SOD mimics are a group of substances which catalyze the oxidation and reduction of O_2_^∙−^. They include cationic metalloporphyrins with Mn porphyrins (MnP), Mn(III) salens, Mn(II) cyclic polyamines, metal oxides, Mn(III) biliverdins, and metallocorroles (MCs) [114]. MnSOD mimics are stoichiometric scavengers of O_2_^∙−^ and accumulate in mitochondria depending on positive charge and lipophilicity [115]. MnPs are among the most potent MnSOD mimics designed and optimized to mimic the action of the enzyme catalytic site and to increase mitochondrial accumulation [115]. Manganese (III) tetrakis(1-methyl-4 pyridyl) porphyrin (MnTMPyP), one of the MnPs, acts as SOD mimic and peroxynitrite scavenger (Figure 4). MnTMPyP decreased lipid peroxidation, nitrotyrosine content in the proximal tubular region, caspase-3 activation, and tubular epithelial cell damage following ischemia/reperfusion [109]. In addition, MnTMPyP decreased the expression of the proapoptotic genes Bax and FasL. At first, the effects of SOD mimics were almost exclusively assigned to the removal of O_2_^∙−^ and ONOO^−^, but recent data suggest the direct H_2_O_2_-driven oxidation of signaling proteins such as NF-*κ*B [114, 116]. This was implied in experimental work of Dorai's group who used Mn(III) meso-tetrakis(N-n-hexylpyridinium-2-yl)porphyrin (MnTnHex-2-PyP^5+^) (Figure 4) [113]. They showed that giving of renoprotective cocktail containing MnTnHex-2-PyP^5+^ to rats 24 h before, at the beginning, and 24 h after kidney IRI ameliorated AKI and induced adaptive response via mild prooxidative stress [113]. The improvement of renoprotective cocktail was achieved by adding N-acetylcysteine that couples with MnTnHex-2-PyP^5+^. As a result, oxidative stress was enhanced via production of H_2_O_2_ [112, 116]. The prooxidative action of MnPs is manifested by oxidation of Cys-62 of p50 subunit of NF-*κ*B, thereby preventing NF-*κ*B activation [116, 117]. It has been proposed that, upon oxidation of cysteines of Kelch-like ECH-associated protein 1 (KEAP1) that is nuclear factor-E2-related factor (Nrf2) inhibitor, porphyrin-based SOD mimics, Mn(II) cyclic polyamines, and nitroxides activate Nrf2. Activation of Nrf2 results in upregulation of endogenous antioxidative defenses [117].

There are several other investigated MnSOD mimics such as MnSalens, mangafodipir (Mn complex with dipyridoxyl diphosphate), substances named tempone and oxazolidine-5-doxylstearate belonging to nitroxides, and MitoSOD, consisting of TTP cation conjugated with a O_2_^∙−^-selective pentaaza macrocyclic Mn(II) SOD mimic, but the data on the use of these compounds* in vivo* kidney ischemia/reperfusion models are still missing [115]. Mito-CP is a five-membered nitroxide CP, conjugated to a TTP cation. It has prevented cisplatin-induced renal dysfunction, renal cell inflammation, and tubular cell apoptosis [83]. Favorable effects in this model of AKI are promising for investigating the role of Mito-CP in renal IRI. Other potential compounds to be investigated in the prevention of kidney IRI include vitamin E [118], lipoic acid [119], and Ebselen [120] conjugated to the TPP cation and targeted to mitochondria. Although 4-hydroxy-2,2,6,6-tetramethylpiperidin-1-oxyl (Tempol) is not SOD mimic, it is a free radical scavenger and has been used in experimental model of kidney IRI. Tempol significantly reduced the increase in creatinine after kidney IRI induction in rats [121, 122]. Also, Tempol significantly reduced kidney MDA level and nitrotyrosine staining.

Overall, these findings support the potential use some of SOD mimics and previously mentioned mitochondria-targeted antioxidants as therapeutic agents in renal IRI (Table 1).

## 5. Conclusion

Mitochondrial ROS generation participates in deleterious cascade of events provoked by ischemia/reperfusion leading to tubular cell death and AKI. So far, investigated treatment by antioxidants has not been successfully translated into clinical practice. Mitochondria-targeted antioxidants represent a novel approach especially in clinical settings in which kidney IRI could be prevented or ameliorated. Experimental data are encouraging despite the fact that most of the data come from the studies exploring myocardial IRI. Those results justify development of this preventative strategy in kidney IRI for clinical use as some of them such as MitoQ and (SS)-31 are already being evaluated in humans for prevention of myocardial IRI.

## Figures and Tables

**Table 1:** Mitochondria-targeted antioxidants in kidney ischemia/reperfusion injury.

Drug	Antioxidant moiety	Reference
MitoQ	Ubiquinone	[72]
SkQ1, SkQR1	Plastoquinone	[74–76]
SS-31 (Bendavia)	Tyr or Dmt (2′,6′-dimethyltyrosine) residues	[106, 107]
SOD mimic		
(i) MnTMPyP	Manganese metalloporphyrin	[109–113]
(ii) MnTnHex-2-PyP^5+^		

## References

[1] Schrier R. W., Wang W., Poole B., Mitra A. (2004). Acute renal failure: definitions, diagnosis, pathogenesis, and therapy. *The Journal of Clinical Investigation*.

[2] Chertow G. M., Burdick E., Honour M., Bonventre J. V., Bates D. W. (2005). Acute kidney injury, mortality, length of stay, and costs in hospitalized patients. *Journal of the American Society of Nephrology*.

[3] Perico N., Cattaneo D., Sayegh M. H., Remuzzi G. (2004). Delayed graft function in kidney transplantation. *The Lancet*.

[4] Boletis J., Balitsari A., Filiopoulos V. (2005). Delayed renal graft function: the influence of immunosuppression. *Transplantation Proceedings*.

[5] Basile D. P. (2007). The endothelial cell in ischemic acute kidney injury: implications for acute and chronic function. *Kidney International*.

[6] Bonventre J. V., Yang L. (2011). Cellular pathophysiology of ischemic acute kidney injury. *The Journal of Clinical Investigation*.

[7] Venkatachalam M. A., Griffin K. A., Lan R., Geng H., Saikumar P., Bidani A. K. (2010). Acute kidney injury: a springboard for progression in chronic kidney disease. *American Journal of Physiology—Renal Physiology*.

[8] Devarajan P. (2006). Update on mechanisms of ischemic acute kidney injury. *Journal of the American Society of Nephrology*.

[9] Ristić A. J., Savić D., Sokić D. (2015). Hippocampal antioxidative system in mesial temporal lobe epilepsy. *Epilepsia*.

[10] Bindoli A., Rigobello M. P. (2013). Principles in redox signaling: from chemistry to functional significance. *Antioxidants & Redox Signaling*.

[11] Andrades M. É., Morina A., Spasić S., Spasojević I. (2011). Bench-to-bedside review: sepsis—from the redox point of view. *Critical Care*.

[12] Klebanoff S. J. (1980). Oxygen metabolism and the toxic properties of phagocytes. *Annals of Internal Medicine*.

[13] Thannickal V. J., Fanburg B. L. (2000). Reactive oxygen species in cell signaling. *American Journal of Physiology—Lung Cellular and Molecular Physiology*.

[14] Ferrer-Sueta G., Radi R. (2009). Chemical biology of peroxynitrite: kinetics, diffusion, and radicals. *ACS Chemical Biology*.

[15] Goldstein S., Czapski G. (2000). Reactivity of ONOO-versus simultaneous generation of ^*∙*^NO and O_2_
^∙−^ toward NADH. *Chemical Research in Toxicology*.

[16] Michelson A. M., Maral J. (1983). Carbonate anions; effects on the oxidation of luminol, oxidative hemolysis, *γ*-irradiation and the reaction of activated oxygen species with enzymes containing various active centres. *Biochimie*.

[17] Chen Q., Moghaddas S., Hoppel C. L., Lesnefsky E. J. (2008). Ischemic defects in the electron transport chain increase the production of reactive oxygen species from isolated rat heart mitochondria. *American Journal of Physiology—Cell Physiology*.

[18] Bajwa A., Kinsey G. R., Okusa M. D. (2009). Immune mechanisms and novel pharmacological therapies of acute kidney injury. *Current Drug Targets*.

[19] Silver S. A., Cardinal H., Colwell K., Burger D., Dickhout J. G. (2015). Acute kidney injury: preclinical innovations, challenges, and opportunities for translation. *Canadian Journal of Kidney Health and Disease*.

[20] Faubel S., Chawla L. S., Chertow G. M., Goldstein S. L., Jaber B. L., Liu K. D. (2012). Ongoing clinical trials in AKI. *Clinical Journal of the American Society of Nephrology*.

[21] Gassanov N., Nia A. M., Caglayan E., Er F. (2014). Remote ischemic preconditioning and renoprotection: from myth to a novel therapeutic option?. *Journal of the American Society of Nephrology*.

[22] Kalogeris T., Bao Y., Korthuis R. J. (2014). Mitochondrial reactive oxygen species: a double edged sword in ischemia/reperfusion vs. preconditioning. *Redox Biology*.

[23] Chen Y.-R., Zweier J. L. (2014). Cardiac mitochondria and reactive oxygen species generation. *Circulation Research*.

[24] Dalton T. P., Shertzer H. G., Puga A. (1999). Regulation of gene expression by reactive oxygen. *Annual Review of Pharmacology and Toxicology*.

[25] Irani K., Xia Y., Zweier J. L. (1997). Mitogenic signaling mediated by oxidants in ras-transformed fibroblasts. *Science*.

[26] Bolli R. (2000). The late phase of preconditioning. *Circulation Research*.

[27] Rouslin W. (1983). Mitochondrial complexes I, II, III, IV, and V in myocardial ischemia and autolysis. *American Journal of Physiology—Heart and Circulatory Physiology*.

[28] Baker J. E., Kalyanaraman B. (1989). Ischemia-induced changes in myocardial paramagnetic metabolites: implications for intracellular oxy-radical generation. *FEBS Letters*.

[29] Mojia M., Pristov J. B., Maksimović-Ivanić D. (2014). Extracellular iron diminishes anticancer effects of vitamin C: an in vitro study. *Scientific Reports*.

[30] Arduini A., Mezzetti A., Porreca E. (1988). Effect of ischemia and reperfusion on antioxidant enzymes and mitochondrial inner membrane proteins in perfused rat heart. *Biochimica et Biophysica Acta (BBA)—Molecular Cell Research*.

[31] Jassem W., Ciarimboli C., Cerioni P. N., Saba V., Norton S. J., Principato G. (1996). Glyoxalase II and glutathione levels in rat liver mitochondria during cold storage in Euro-Collins and University of Wisconsin solutions. *Transplantation*.

[32] Whitmarsh A. J., Davis R. J. (1996). Transcription factor AP-1 regulation by mitogen-activated protein kinase signal transduction pathways. *Journal of Molecular Medicine*.

[33] McGowan A. J., Ruiz-Ruiz M. C., Gorman A. M., Lopez-Rivas A., Cotter T. G. (1996). Reactive oxygen intermediate(s) (ROI): common mediator(s) of poly(ADP-ribose)polymerase (PARP) cleavage and apoptosis. *FEBS Letters*.

[34] Latanich C. A., Toledo-Pereyra L. H. (2009). Searching for NF-*κ*B-based treatments of ischemia reperfusion injury. *Journal of Investigative Surgery*.

[35] Nichols T. C. (2004). NF-*κ*B and reperfusion injury. *Drug News and Perspectives*.

[36] Cao C. C., Ding X. Q., Ou Z. L. (2004). In vivo transfection of NF-*κ*B decoy oligodeoxynucleotides attenuate renal ischemia/reperfusion injury in rats. *Kidney International*.

[37] Kezic A., Becker J. U., Thaiss F. (2013). The effect of mTOR-inhibition on NF-*κ*B activity in kidney ischemia-reperfusion injury in mice. *Transplantation Proceedings*.

[38] Wan X., Fan L., Hu B. (2011). Small interfering RNA targeting IKK*β* prevents renal ischemia-reperfusion injury in rats. *American Journal of Physiology. Renal Physiology*.

[39] Rius J., Guma M., Schachtrup C. (2008). NF-*κ*B links innate immunity to the hypoxic response through transcriptional regulation of HIF-1*α*. *Nature*.

[40] Lee H.-L., Chen C.-L., Yeh S. T., Zweier J. L., Chen Y.-R. (2012). Biphasic modulation of the mitochondrial electron transport chain in myocardial ischemia and reperfusion. *American Journal of Physiology—Heart and Circulatory Physiology*.

[41] Wang P., Zweier J. L. (1996). Measurement of nitric oxide and peroxynitrite generation in the postischemic heart: evidence for peroxynitrite-mediated reperfusion injury. *Journal of Biological Chemistry*.

[42] Saito M., Miyagawa I. (2000). Real-time monitoring of nitric oxide in ischemia-reperfusion rat kidney. *Urological Research*.

[43] Salom M. G., Arregui B., Carbonell L. F., Ruiz F., González-Mora J. L., Fenoy F. J. (2005). Renal ischemia induces an increase in nitric oxide levels from tissue stores. *American Journal of Physiology—Regulatory Integrative and Comparative Physiology*.

[44] Walker L. M., York J. L., Imam S. Z., Ali S. F., Muldrew K. L., Mayeux P. R. (2001). Oxidative stress and reactive nitrogen species generation during renal ischemia. *Toxicological Sciences*.

[45] Noiri E., Peresleni T., Miller F., Goligorsky M. S. (1996). In vivo targeting of inducible NO synthase with oligodeoxynucleotides protects rat kidney against ischemia. *The Journal of Clinical Investigation*.

[46] Chiao H., Kohda Y., McLeroy P., Craig L., Linas S., Star R. A. (1998). *α*-Melanocyte-stimulating hormone inhibits renal injury in the absence of neutrophils. *Kidney International*.

[47] Chatterjee P. K., Patel N. S. A., Kvale E. O. (2002). Inhibition of inducible nitric oxide synthase reduces renal ischemia/reperfusion injury. *Kidney International*.

[48] Kezic A., Thaiss F., Becker J. U., Tsui T. Y., Bajcetic M. (2013). Effects of everolimus on oxidative stress in kidney model of ischemia/reperfusion injury. *American Journal of Nephrology*.

[49] Ling H., Gengaro P. E., Edelstein C. L. (1998). Effect of hypoxia on proximal tubules isolated from nitric oxide synthase knockout mice. *Kidney International*.

[50] Zorov D. B., Juhaszova M., Sollott S. J. (2006). Mitochondrial ROS-induced ROS release: an update and review. *Biochimica et Biophysica Acta (BBA)—Bioenergetics*.

[51] Halestrap A. P., Kerr P. M., Javadov S., Woodfield K.-Y. (1998). Elucidating the molecular mechanism of the permeability transition pore and its role in reperfusion injury of the heart. *Biochimica et Biophysica Acta—Bioenergetics*.

[52] Crompton M. (1999). The mitochondrial permeability transition pore and its role in cell death. *The Biochemical Journal*.

[53] Kelso G. F., Porteous C. M., Coulter C. V. (2001). Selective targeting of a redox-active ubiquinone to mitochondria within cells: antioxidant and antiapoptotic properties. *The Journal of Biological Chemistry*.

[54] Skulachev V. P., Anisimov V. N., Antonenko Y. N. (2009). An attempt to prevent senescence: a mitochondrial approach. *Biochimica et Biophysica Acta—Bioenergetics*.

[55] Cochemé H. M., Kelso G. F., James A. M. (2007). Mitochondrial targeting of quinones: therapeutic implications. *Mitochondrion*.

[56] Murphy M. P. (1997). Selective targeting of bioactive compounds to mitochondria. *Trends in Biotechnology*.

[57] Yousif L. F., Stewart K. M., Kelley S. O. (2009). Targeting mitochondria with organelle-specific compounds: strategies and applications. *ChemBioChem*.

[58] Szeto H. H. (2006). Mitochondria-targeted peptide antioxidants: novel neuroprotective agents. *The AAPS Journal*.

[59] Zhao K., Zhao G.-M., Wu D. (2004). Cell-permeable peptide antioxidants targeted to inner mitochondrial membrane inhibit mitochondrial swelling, oxidative cell death, and reperfusion injury. *Journal of Biological Chemistry*.

[60] Winterbourn C. C., Parsons-Mair H. N., Gebicki S., Gebicki J. M., Davies M. J. (2004). Requirements for superoxide-dependent tyrosine hydroperoxide formation in peptides. *Biochemical Journal*.

[61] Asayama S., Kawamura E., Nagaoka S., Kawakami H. (2006). Design of manganese porphyrin modified with mitochondrial signal peptide for a new antioxidant. *Molecular Pharmaceutics*.

[62] Hanly L. N., Chen N., Aleksa K. (2012). N-acetylcysteine as a novel prophylactic treatment for ifosfamide-induced nephrotoxicity in children: translational pharmacokinetics. *Journal of Clinical Pharmacology*.

[63] Sheu S.-S., Nauduri D., Anders M. W. (2006). Targeting antioxidants to mitochondria: a new therapeutic direction. *Biochimica et Biophysica Acta—Molecular Basis of Disease*.

[64] Gross G. J., Auchampach J. A. (1990). Blockade of ATP-sensitive potassium channels prevents myocardial preconditioning in dogs. *Circulation Research*.

[65] Szewczyk A., Jarmuszkiewicz W., Kunz W. S. (2009). Mitochondrial potassium channels. *IUBMB Life*.

[66] Snow B. J., Rolfe F. L., Lockhart M. M. (2010). A double-blind, placebo-controlled study to assess the mitochondria-targeted antioxidant MitoQ as a disease-modifying therapy in Parkinson's disease. *Movement Disorders*.

[67] Gane E. J., Weilert F., Orr D. W. (2010). The mitochondria-targeted anti-oxidant mitoquinone decreases liver damage in a phase II study of hepatitis C patients. *Liver International*.

[68] James A. M., Cochemé H. M., Smith R. A. J., Murphy M. P. (2005). Interactions of mitochondria-targeted and untargeted ubiquinones with the mitochondrial respiratory chain and reactive oxygen species: implications for the use of exogenous ubiquinones as therapies and experimental tools. *The Journal of Biological Chemistry*.

[69] Mukhopadhyay P., Horváth B., Zsengellėr Z. (2012). Mitochondrial reactive oxygen species generation triggers inflammatory response and tissue injury associated with hepatic ischemia–reperfusion: therapeutic potential of mitochondrially targeted antioxidants. *Free Radical Biology and Medicine*.

[70] Adlam V. J., Harrison J. C., Porteous C. M. (2005). Targeting an antioxidant to mitochondria decreases cardiac ischemia-reperfusion injury. *The FASEB Journal*.

[71] Mitchell T., Rotaru D., Saba H., Smith R. A. J., Murphy M. P., MacMillan-Crow L. A. (2011). The mitochondria-targeted antioxidant mitoquinone protects against cold storage injury of renal tubular cells and rat kidneys. *Journal of Pharmacology and Experimental Therapeutics*.

[72] Dare A. J., Bolton E. A., Pettigrew G. J., Bradley J. A., Saeb-Parsy K., Murphy M. P. (2015). Protection against renal ischemia-reperfusion injury in vivo by the mitochondria targeted antioxidant MitoQ. *Redox Biology*.

[73] Antonenko Y. N., Avetisyan A. V., Bakeeva L. E. (2008). Mitochondria-targeted plastoquinone derivatives as tools to interrupt execution of the aging program. 1. Cationic plastoquinone derivatives: synthesis and *in vitro* studies. *Biochemistry*.

[74] Bakeeva L. E., Barskov I. V., Egorov M. V. (2008). Mitochondria-targeted plastoquinone derivatives as tools to interrupt execution of the aging program. 2. Treatment of some ROS- and age-related diseases (heart arrhythmia, heart infarctions, kidney ischemia, and stroke). *Biochemistry*.

[75] Plotnikov E. Y., Vasileva A. K., Arkhangelskaya A. A., Pevzner I. B., Skulachev V. P., Zorov D. B. (2008). Interrelations of mitochondrial fragmentation and cell death under ischemia/reoxygenation and UV-irradiation: protective effects of SkQ1, lithium ions and insulin. *FEBS Letters*.

[76] Plotnikov E. Y., Chupyrkina A. A., Jankauskas S. S. (2011). Mechanisms of nephroprotective effect of mitochondria-targeted antioxidants under rhabdomyolysis and ischemia/reperfusion. *Biochimica et Biophysica Acta (BBA)—Molecular Basis of Disease*.

[77] Sharples E. J., Yaqoob M. M. (2006). Erythropoietin in experimental acute renal failure. *Nephron Experimental Nephrology*.

[78] Sharples E. J., Patel N., Brown P. (2004). Erythropoietin protects the kidney against the injury and dysfunction caused by ischemia-reperfusion. *Journal of the American Society of Nephrology*.

[79] Song Y. R., Lee T., You S. J. (2009). Prevention of acute kidney injury by erythropoietin in patients undergoing coronary artery bypass grafting: a pilot study. *American Journal of Nephrology*.

[80] Juhaszova M., Zorov D. B., Kim S. H. (2004). Glycogen synthase kinase- 3beta mediates convergence of protection signaling to inhibit the mitochondrial permeability transition pore. *Journal of Clinical Investigation*.

[81] Wang Z., Ge Y., Bao H., Dworkin L., Peng A., Gong R. (2013). Redox-sensitive glycogen synthase kinase 3*β*-directed control of mitochondrial permeability transition: rheostatic regulation of acute kidney injury. *Free Radical Biology and Medicine*.

[82] Plotnikov E. Y., Kazachenko A. V., Vyssokikh M. Y. (2007). The role of mitochondria in oxidative and nitrosative stress during ischemia/reperfusion in the rat kidney. *Kidney International*.

[83] Mukhopadhyay P., Horváth B., Zsengellér Z. (2012). Mitochondrial-targeted antioxidants represent a promising approach for prevention of cisplatin-induced nephropathy. *Free Radical Biology and Medicine*.

[84] Jankauskas S. S., Plotnikov E. Y., Morosanova M. A. (2012). Mitochondria-targeted antioxidant SkQR1 ameliorates gentamycin-induced renal failure and hearing loss. *Biochemistry*.

[85] Facundo H. T. F., de Paula J. G., Kowaltowski A. J. (2005). Mitochondrial ATP-sensitive K^+^ channels prevent oxidative stress, permeability transition and cell death. *Journal of Bioenergetics and Biomembranes*.

[86] Facundo H. T. F., de Paula J. G., Kowaltowski A. J. (2007). Mitochondrial ATP-sensitive K^+^ channels are redox-sensitive pathways that control reactive oxygen species production. *Free Radical Biology and Medicine*.

[87] Domoki F., Bari F., Nagy K., Busija D. W., Siklós L. (2004). Diazoxide prevents mitochondrial swelling and Ca^2+^ accumulation in CA1 pyramidal cells after cerebral ischemia in newborn pigs. *Brain Research*.

[88] Ichinose M., Yonemochi H., Sato T., Saikawa T. (2003). Diazoxide triggers cardioprotection against apoptosis induced by oxidative stress. *American Journal of Physiology—Heart and Circulatory Physiology*.

[89] Reeves W. B., Shah S. V. (1994). Activation of potassium channels contributes to hypoxic injury in proximal tubules. *The Journal of Clinical Investigation*.

[90] Sun Z., Zhang X., Ito K. (2008). Amelioration of oxidative mitochondrial DNA damage and deletion after renal ischemic injury by the KATP channel opener diazoxide. *American Journal of Physiology—Renal Physiology*.

[91] Javadov S., Karmazyn M., Escobales N. (2009). Mitochondrial permeability transition pore opening as a promising therapeutic target in cardiac diseases. *Journal of Pharmacology and Experimental Therapeutics*.

[92] Morin D., Assaly R., Paradis S., Berdeaux A. (2009). Inhibition of mitochondrial membrane permeability as a putative pharmacological target for cardioprotection. *Current Medicinal Chemistry*.

[93] Devalaraja-Narashimha K., Diener A. M., Padanilam B. J. (2009). Cyclophilin D gene ablation protects mice from ischemic renal injury. *American Journal of Physiology—Renal Physiology*.

[94] Yang C. W., Ahn H. J., Han H. J. (2001). Pharmacological preconditioning with low-dose cyclosporine or FK506 reduces subsequent ischemia/reperfusion injury in rat kidney. *Transplantation*.

[95] Singh D., Chander V., Chopra K. (2005). Cyclosporine protects against ischemia/reperfusion injury in rat kidneys. *Toxicology*.

[96] Ysebaert D. K., De Greef K. E., Nouwen E. J., Verpooten G. A., Eyskens E. J., De Broe M. E. (1997). Influence of cyclosporin A on the damage and regeneration of the kidney after severe ischemia/reperfusion injury. *Transplantation Proceedings*.

[97] Gonçalves G. M., Cenedeze M. A., Feitoza C. Q. (2007). The role of immunosuppressive drugs in aggravating renal ischemia and reperfusion injury. *Transplantation Proceedings*.

[98] Zhao K., Zhao G.-M., Wu D. (2004). Cell-permeable peptide antioxidants targeted to inner mitochondrial membrane inhibit mitochondrial swelling, oxidative cell death, and reperfusion injury. *The Journal of Biological Chemistry*.

[99] Brown D. A., Sabbah H. N., Shaikh S. R. (2013). Mitochondrial inner membrane lipids and proteins as targets for decreasing cardiac ischemia/reperfusion injury. *Pharmacology and Therapeutics*.

[100] Dai D.-F., Chen T., Szeto H. (2011). Mitochondrial targeted antioxidant peptide ameliorates hypertensive cardiomyopathy. *Journal of the American College of Cardiology*.

[101] Yang L., Zhao K., Calingasan N. Y., Luo G., Szeto H. H., Beal M. F. (2009). Mitochondria targeted peptides protect against 1-methyl-4-phenyl-1,2,3,6-tetrahydropyridine neurotoxicity. *Antioxidants & Redox Signaling*.

[102] Cho S., Szeto H. H., Kim E., Kim H., Tolhurst A. T., Pinto J. T. (2007). A novel cell-permeable antioxidant peptide, SS31, attenuates ischemic brain injury by down-regulating CD36. *Journal of Biological Chemistry*.

[103] Kloner R. A., Hale S. L., Dai W. (2012). Reduction of ischemia/reperfusion injury with bendavia, a mitochondria-targeting cytoprotective peptide. *Journal of the American Heart Association*.

[104] Brown D. A., Hale S. L., Baines C. P. (2014). Reduction of early reperfusion injury with the mitochondria-targeting peptide bendavia. *Journal of Cardiovascular Pharmacology and Therapeutics*.

[105] Chakrabarti A. K., Feeney K., Abueg C. (2013). Rationale and design of the EMBRACE STEMI Study: a phase 2a, randomized, double-blind, placebo-controlled trial to evaluate the safety, tolerability and efficacy of intravenous Bendavia on reperfusion injury in patients treated with standard therapy including primary percutaneous coronary intervention and stenting for ST-segment elevation myocardial infarction. *American Heart Journal*.

[106] Szeto H. H., Liu S., Soong Y. (2011). Mitochondria-targeted peptide accelerates ATP recovery and reduces ischemic kidney injury. *Journal of the American Society of Nephrology*.

[107] Eirin A., Li Z., Zhang X. (2012). A mitochondrial permeability transition pore inhibitor improves renal outcomes after revascularization in experimental atherosclerotic renal artery stenosis. *Hypertension*.

[108] Parajuli N., MacMillan-Crow L. A. (2013). Role of reduced manganese superoxide dismutase in ischemia-reperfusion injury: a possible trigger for autophagy and mitochondrial biogenesis?. *American Journal of Physiology—Renal Physiology*.

[109] Liang H. L., Hilton G., Mortensen J., Regner K., Johnson C. P., Nilakantan V. (2009). MnTMPyP, a cell-permeant SOD mimetic, reduces oxidative stress and apoptosis following renal ischemia-reperfusion. *American Journal of Physiology—Renal Physiology*.

[110] Dobashi K., Ghosh B., Orak J. K., Singh I., Singh A. K. (2000). Kidney ischemia-reperfusion: modulation of antioxidant defenses. *Molecular and Cellular Biochemistry*.

[111] Saba H., Batinic-Haberle I., Munusamy S. (2007). Manganese porphyrin reduces renal injury and mitochondrial damage during ischemia/reperfusion. *Free Radical Biology and Medicine*.

[112] Cohen J., Dorai T., Ding C., Batinic-Haberle I., Grasso M. (2013). The administration of renoprotective agents extends warm ischemia in a rat model. *Journal of Endourology*.

[113] Dorai T., Fishman A. I., Ding C., Batinic-Haberle I., Goldfarb D. S., Grasso M. (2011). Amelioration of renal ischemia-reperfusion injury with a novel protective cocktail. *Journal of Urology*.

[114] Batinic-Haberle I., Tovmasyan A., Roberts E. R. H., Vujaskovic Z., Leong K. W., Spasojevic I. (2014). SOD therapeutics: latest insights into their structure-activity relationships and impact on the cellular redox-based signaling pathways. *Antioxidants & Redox Signaling*.

[115] Miriyala S., Spasojevic I., Tovmasyan A. (2012). Manganese superoxide dismutase, MnSOD and its mimics. *Biochimica et Biophysica Acta*.

[116] Batinic-Haberle I., Spasojevic I., Tse H. M. (2012). Design of Mn porphyrins for treating oxidative stress injuries and their redox-based regulation of cellular transcriptional activities. *Amino Acids*.

[117] Batinic-Haberle I., Tovmasyan A., Spasojevic I. (2015). An educational overview of the chemistry, biochemistry and therapeutic aspects of Mn porphyrins—from superoxide dismutation to H_2_O_2_-driven pathways. *Redox Biology*.

[118] Smith R. A. J., Porteous C. M., Coulter C. V., Murphy M. P. (1999). Selective targeting of an antioxidant to mitochondria. *European Journal of Biochemistry*.

[119] Brown S. E., Ross M. F., Sanjuan-Pla A., Manas A.-R. B., Smith R. A. J., Murphy M. P. (2007). Targeting lipoic acid to mitochondria: synthesis and characterization of a triphenylphosphonium-conjugated *α*-lipoyl derivative. *Free Radical Biology and Medicine*.

[120] Filipovska A., Kelso G. F., Brown S. E., Beer S. M., Smith R. A. J., Murphy M. P. (2005). Synthesis and characterization of a triphenylphosphonium-conjugated peroxidase mimetic: insights into the interaction of ebselen with mitochondria. *The Journal of Biological Chemistry*.

[121] Chatterjee P. K., Cuzzocrea S., Brown P. A. J. (2000). Tempol, a membrane-permeable radical scavenger, reduces oxidant stress-mediated renal dysfunction and injury in the rat. *Kidney International*.

[122] Aksu U., Ergin B., Bezemer R. (2015). Scavenging reactive oxygen species using tempol in the acute phase of renal ischemia/reperfusion and its effects on kidney oxygenation and nitric oxide levels. *Intensive Care Medicine Experimental*.

